# MAIT cells and viruses

**DOI:** 10.1111/imcb.12008

**Published:** 2018-02-10

**Authors:** James E Ussher, Christian B Willberg, Paul Klenerman

**Affiliations:** ^1^ Microbiology and Immunology University of Otago Dunedin New Zealand; ^2^ Peter Medawar Building for Pathogen Research Nuffield Department of Medicine University of Oxford Oxford UK; ^3^ NIHR Biomedical Research Centre Oxford UK; ^4^ Translational Gastroenterology Unit Oxford University Hospitals Oxford UK

**Keywords:** cytokines, MAIT cells, MR1, TLR, virus

## Abstract

Mucosal associated invariant T cells (MAIT cells) bear a T cell receptor (TCR) that specifically targets microbially derived metabolites. Functionally, they respond to bacteria and yeasts, which possess the riboflavin pathway, essential for production of such metabolites and which are presented on MR1. Viruses cannot generate these ligands, so *a priori*, they should not be recognized by MAIT cells and indeed this is true when considering recognition through the TCR. However, MAIT cells are distinctive in another respect, since they respond quite sensitively to non‐TCR signals, especially in the form of inflammatory cytokines. Thus, a number of groups have shown that virus infection can be “sensed” by MAIT cells and a functional response invoked. Since MAIT cells are abundant in humans, especially in tissues such as the liver, the question has arisen as to whether this TCR‐independent MAIT cell triggering by viruses plays any role *in vivo*. In this review, we will discuss the evidence for this phenomenon and some common features which emerge across different recent studies in this area.

## TCR‐Independent Activation of MAIT Cells

Mucosal associated invariant T (MAIT) cells are antibacterial T cells with a semi‐invariant TCR (Vα7.2‐Jα12/20/33),^1^, ^2^, ^3^, ^4^ restricted by the nonpolymorphic, highly evolutionarily conserved, MHC class Ib molecule, MR1.^1^, ^5^ MAIT cells were first described over 20 years ago, but it is only recently that they have gained much attention, as is described by the review of Olivier Lantz in this collection. The discovery that they responded to bacteria, and the subsequent discovery of the ligand by Rossjohn and McCluskey groups has led to a flurry of activity in the last few years.^6^, ^7^ It is now well recognized that MR1 presents unstable pyrimidine intermediates derived from the riboflavin biosynthesis pathway,^6^, ^7^ a pathway that is present in most bacteria and some fungi, but not in mice or humans.^7^, ^8^ While there is speculation that other ligands exist,^9^ including endogenous ligands,^10^ a viral ligand for MR1 is yet to be described.

Some groups also identified this cell type in human studies as an abundant and clearly identifiable CD8^+^ T cell population with distinct functions, marked by high level expression of the C‐type lectin CD161, but without definition of the TCR.^11^, ^12^ In adult humans, the vast majority of the CD161^++^ (or bright) CD8^+^ T cell population are MAIT cells as defined by Vα7.2 expression or MR1 tetramer binding, although the situation is quite different in cord blood and in young infants, where the TCR usage is much more mixed.^4^, ^13^, ^14^ For the purposes of this review, we are considering a virus‐responsive behavior which is common among CD161^++^ CD8^+^ T cell populations, including tightly defined MAIT cells; we are referring to MAIT cells isolated from blood unless otherwise stated. To some extent, this recapitulates how the data emerged. It still holds true that this population as a whole hase conserved characteristics, which also extend into related CD161‐expressing innate T cell populations (see below).^15^, ^16^ It is also the case that such MAIT cell functionality appears to be more or less identical when comparing the CD8^+^ and the CD8^−^CD4^−^ double negative population of MAIT cells (which also express high levels of CD161).^17^

**Figure 1:**
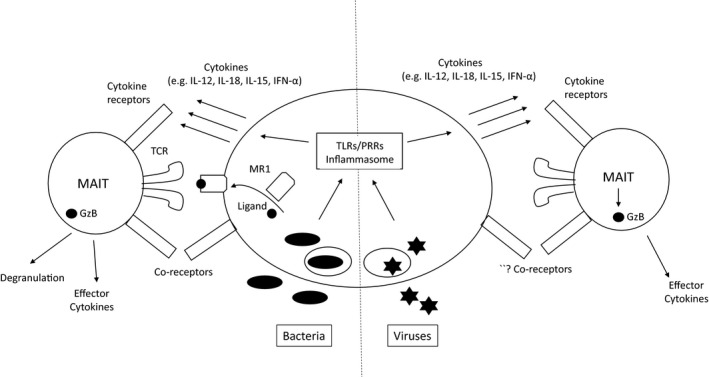
Activation of MAIT cells in a TCR‐dependent *versus* TCR‐independent manner. The left‐hand side shows activation following bacterial stimulation in the case of a bacterial species which has the riboflavin operon active and can produce the ligand—these may be cytosolic or taken up into phagosomes in a professional antigen‐presenting cell. Recognition occurs via TCR through recognition of ligand‐MR1 complexes, accompanied by cell surface signals (such as CD28/CD80 interactions) and cytokines. The balance between MR1‐dependent and ‐independent signals may vary over time, even if ligand is produced. The right‐hand side shows the situation in response to viruses, where ligand is not present, but cytokines will be produced through triggering of toll‐like receptors (TLRs) or other pattern recognition receptors (PRRs) which also lead to inflammasome activation. The role of coreceptor stimulation in this setting is unknown. In both cases, granzyme B activation is seen, although whether this can lead to degranulation in the setting of virus infection is not known.

MAIT cells can be activated by cytokines independently of their TCR (Figure 1). MAIT cells express high levels of the interleukin (IL) 18 receptor (IL‐18R), significantly higher than other blood lymphocyte populations, and also express the IL‐12R.^3^, ^11^, ^12^, ^18^, ^19^, ^20^ It is known that IL‐18 can synergize with IL‐12 to stimulate interferon‐γ production by human T cells, natural killer (NK) cells and murine T cells, including NKT cells.^21^, ^22^, ^23^, ^24^ Similarly, stimulation of MAIT cells with IL‐12 and IL‐18 resulted in production of interferon‐γ.^18^ Of note, in human peripheral blood mononuclear cells (PBMC), MAIT cells were the predominant source of IL‐12‐ and IL‐18‐induced interferon‐γ production, although other CD161‐expressing T cell populations (CD8^+^, CD4^+^ and γδ T cells) share the same capability.^16^, ^18^ Interferon‐γ production required stimulation with both cytokines; neither on its own was able to stimulate significant interferon‐γ production.^18^ IL‐12 and IL‐18 also stimulated production of TNFα, although to a lesser extent, and upregulation of granzyme B and CD69.^25^ IL‐12‐ and IL‐18‐stimulated interferon‐γ production was not affected by MR1 blockade or inhibition of TCR signaling, but was dependent upon p38‐MAPK signaling.^18^ The kinetics of interferon‐γ production in response to IL‐12 and IL‐18 differs from T cell receptor stimulation: when stimulated with *Escherichia coli*, rapid production of interferon‐γ was seen (within hours) that was dependent upon MR1; in contrast, activation at 20 h post stimulation was dependent upon both MR1 and cytokines.^18^ Liver‐resident MAIT cells also express IL‐12R and IL‐18R, and produce interferon‐γ in response to IL‐12 and IL‐18; a similar time course of activation was seen.^20^, ^26^ While TCR‐mediated stimulation is more rapid, it is also more transient, as was recently shown when isolated MAIT cells were stimulated with anti‐CD3/CD28 beads or cytokines.^27^

Other cytokines can also activate MAIT cells in the absence of TCR stimulation. Stimulation of PBMC with IL‐15 resulted in interferon‐γ production, and upregulation of CD69, granzyme B, perforin and T‐bet.^19^ This was dependent upon production of IL‐18‐ by IL‐15‐stimulated monocytes. While MAIT cells express the IL‐15 receptor, IL‐15 failed to directly activate sort‐purified MAIT cells. When PBMC were treated with IL‐15, MAIT cell activation could be inhibited by blocking either IL‐15 or IL‐18. Activation in response to IL‐15 was slower than to IL‐12 and IL‐18, with activation seen at 36 h but little at 18 h. Activation was also seen with the common gamma chain cytokines IL‐2 and IL‐7, but to a lesser degree than with IL‐15.^19^ IL‐12 can also synergize with IL‐15 to activate MAIT cells.^25^ The combination of IL‐12, IL‐15 and IL‐18 led to the activation of most MAIT cells, with more than 80% of cells producing interferon‐γ after 24 h stimulation.^25^

MAIT cells can also be activated by IL‐7. Treatment of PBMC with IL‐7 for 48–60 h increased T cell receptor and CD69 expression, and MAIT cell cytotoxic capacity, with increased expression of perforin, and granzyme A and B.^28^, ^29^ In contrast, treatment of enriched CD8^+^ T cells, containing MAIT cells, for 24 h with IL‐7 resulted in upregulation of perforin but had no effect on cytokine production or granzyme B, A or K expression; similar changes were seen with IL‐2 and IL‐15.^30^ Treatment with IL‐7 for 48–60 h also increased MAIT cell expression of the transcription factors PLZF, RORγ, T‐bet, Eomes and Helios.^29^ While no degranulation or interferon‐γ production was seen with IL‐7 treatment alone, IL‐7 enhanced subsequent responses to T cell receptor stimulation; increased granzyme B, degranulation, cytotoxicity, and production of interferon‐γ and IL‐17A production was seen.^28^, ^29^, ^31^

Type I interferons (α and β), when combined with IL‐12 or IL‐18, can also activate MAIT cells in the absence of TCR stimulation.^25^ Similar to IL‐12, IL‐15 and IL‐18, type I interferons alone failed to stimulate interferon‐γ production by MAIT cells. In contrast, granzyme B upregulation was seen with IL‐12 or IL‐15 alone, and upregulation of CD69 and production of TNFα was seen with IL‐15 alone, albeit at lower levels than with combinations of cytokines.^25^ IL‐12 alone increased T‐bet expression, while IL‐1β decreased granzyme B and perforin expression.^32^ Future experiments should address whether type I interferons activate MAIT cells directly or indirectly.

Cytokines can also stimulate MAIT proliferation. T cell receptor signaling alone is insufficient for MAIT cell proliferation; costimulation via CD28 or with cytokines (IL‐1β, IL‐12, IL‐18, IL‐23, IL‐2 + IL‐21, IL‐12 + IL‐18) is required.^12^, ^32^, ^33^ Cytokines alone can also stimulate MAIT cell proliferation. Proliferation of MAIT cells was seen after treatment for 6–7 days with IL‐2, IL‐7, IL‐12 or IL‐15 alone.^12^, ^30^, ^33^ No synergy was seen when cytokines were combined.^30^ IL‐18 does not appear to stimulate MAIT cell proliferation.^30^

Cytokine‐treated MAIT cells are also able to provide help to B cells. In a 7‐day coculture of MAIT cells and B cells, treatment with IL‐12 in combination with either IL‐2 or IL‐15 induced antibody production (IgA, IgG and IgM) and the expansion of CD38^+^CD24^−^ plasmablasts; in the absence of MAIT cells, cytokines had minimal effect on B cells.^34^ When cocultures were treated with a single cytokine (IL‐2, IL‐12 or IL‐15 alone), the effect on B cells was lost. In the presence of T cell receptor stimulation (with anti‐CD3/CD28), the addition of IL‐12 and IL‐18 enhanced IgM production and the expansion of plasmablasts in MAIT cell and B cell cocultures; the combination of IL‐12 and IL‐18 had little effect in the absence of T cell receptor signaling.^34^ When sorted B cell populations were cultured with the supernatant from bacterial‐stimulated MAIT cells, plasmablasts were found to derive from switched (IgD‐) and nonswitched (IgD+) CD27^+^ memory B cells^34^; while not assessed, it is reasonable to speculate that the same cell types differentiate into plasmablasts in the presence of cytokine‐stimulated MAIT cells.

A recent report suggests that different subpopulations of MAIT cells respond differently to cytokine stimulation. MAIT cells expressing innate receptors CD56, CD84 and CD94 produced more interferon‐γ in response to stimulation for 24 h with IL‐12 and IL‐18.^35^ CD56^+^ MAIT cells expressed higher levels of IL‐12R, IL‐18R and perforin, as well as the transcription factors PLZF, Eomes and T‐bet. There may be a positive feedback loop as the percentage of MAIT cells expressing CD94 increased with IL‐12 and IL‐18 stimulation.^35^ Additionally, functional differences may also be observed in the relatively rare CD4^+^ MAIT cell subset.^17^

**Figure 2:**
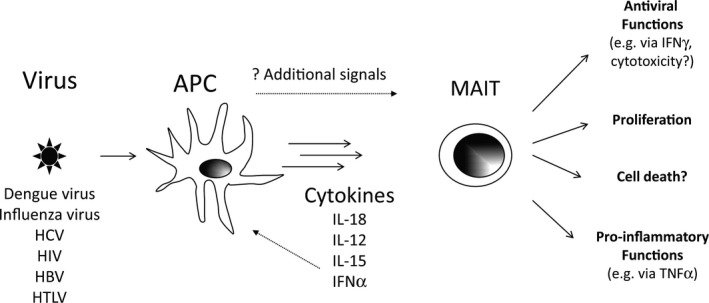
A cartoon illustrating the activation of MAIT cells by viruses. Viruses signal via PRRs to stimulate the production of cytokines. Other signals may also be involved. The exact blend of cytokines involved and the kinetics of their release varies between individual viruses and also between different APCs. Cytokines may activate MAIT cells directly via their cytokine receptors (e.g. IL‐12 and IL‐18) or indirectly through stimulating the release of other cytokines (e.g. IL‐15). Cytokine‐activated MAIT cells may have antiviral functions (possibly including cytotoxicity, although that is yet to be demonstrated), enhance inflammation and proliferate. In chronic viral infections, cytokine‐activated MAIT cells may also undergo activation‐induced cell death.

Overall, MAIT cells can be stimulated independently of their T cell receptor by a number of different cytokines, resulting in MAIT cell activation and proliferation (Figure 2). The exact effector functions of cytokine‐stimulated MAIT cells may differ, however, depending upon the nature of the stimulus.

## Activation of MAIT Cells Via Sensing of TLR Stimuli

Toll‐like‐receptor ligands can activate MAIT cells via induction of activating cytokines. This was first demonstrated by treating PBMC with the TLR8 agonist, ssRNA40.^18^ Production of interferon‐γ by MAIT cells was seen and could not be blocked by an MR1 blocking antibody. In contrast, TLR8‐mediated MAIT cell activation could be blocked by antibodies that neutralized IL‐12 or IL‐18. Inhibiting TLR8 signaling by blocking endosomal acidification or preventing IL‐18 production by inhibiting the inflammasome also inhibited MAIT cell activation.^18^

The same TLR8 agonist also stimulated interferon‐γ production by liver‐derived mononuclear cells.^26^ MAIT cells and CD56^Bright^ NK cells were the predominant source of interferon‐γ. Again, this was dependent upon IL‐12 and IL‐18 production which was produced by monocytes. Most MAIT cells produced interferon‐γ in response to the TLR8 agonist, while a small number also produced TNFα. *Enterococus faecalis,* a nonligand producing bacteria, was also able to stimulate interferon‐γ production from both liver‐derived and blood MAIT cells in an IL‐12 and IL‐18 dependent fashion.^18^, ^26^

Other TLR agonists can also stimulate MAIT cells in a cytokine‐dependent fashion. In PBMC, while weaker than TLR8 agonists, TLR3, and to a lesser extent, TLR2, 4 and 5 agonists, were also able to stimulate some interferon‐γ production.^18^, ^25^ When THP1 cells were used as the antigen‐presenting cell, the TLR4 agonist, lipopolysaccharide, but not other TLR agonists, was able to stimulate interferon‐γ production by MAIT cells.^18^ In a recent paper, monocytes pretreated with a TLR8 agonist or a TLR4 agonist were shown to activate purified MAIT cells (as determined by granzyme B and interferon‐γ expression) in the absence of TCR stimulation; this was not dependent upon cell‐to‐cell contact as the supernatant of TLR8‐treated monocytes had a similar effect. Interestingly, little IL‐12 and no IL‐15 or IL‐18 was detected in cell supernatants, suggesting that other inflammatory cytokines can activate MAIT cells.^27^

Interestingly, there are differences between the effects of TLR agonists on cytokine‐mediated MAIT cell activation, MR1 surface expression and T cell receptor‐mediated MAIT cell activation. Increased surface expression of MR1 in the absence of its pyrimidine intermediate ligand has been seen in THP1 cells stimulated with agonists of TLR2, TLR4 or TLR5.^36^, ^37^ In contrast, TLR1, 2 and 6 agonists, but not the TLR4 agonist lipopolysaccharide, enhanced MR1‐mediated MAIT cell activation in response to *E. coli*.^36^ In primary monocytes, pretreatment with lipopolysaccharide inhibited subsequent MAIT cell activation in response to *E. coli*.^36^ In contrast, pretreatment of primary monocytes with TLR4 or TLR8 agonists enhanced the activation of MAIT cells stimulated with anti‐CD3/CD28 beads.^27^ Recently, the TLR9 agonist CpG was shown to increase MR1 surface expression on B cells, with a small increase also seen on NK cells and monocytes, in the absence of the MR1 pyrimidine intermediate ligand.^37^ Knocking down the expression of TLR9 with shRNA in a B cell line decreased MR1 surface expression and the cells’ ability to stimulate MAIT cells.^37^ TLR ligands also impact MAIT cell function *in vivo*. In a mouse model, intrapulmonary coadministration of TLR ligands Pam2Cys, CpG or polyI:C, or the nonligand producing *Salmonella typhimurium ΔribDH*, with the MR1 ligand 5‐(2‐oxopropylideneamino)‐6‐d‐ribitylaminouracil (5‐OP‐RU), enhanced the accumulation and activation of MAIT cells in the lungs.^38^ In contrast, the TLR ligands and *Salmonella typhimurium ΔribDH* had no effect in the absence of 5‐OP‐RU.^38^ Therefore, the effect of different TLR agonists on MAIT cell activation is likely to depend upon the antigen presenting cell, the range of TLRs that it expresses, the amount of IL‐12 and IL‐18 production induced, and the presence or absence of the MR1 ligand. Different TLR agonists are likely to have different effects on T cell receptor‐dependent and ‐independent MAIT cell activation.

## Activation by Virally Infected Antigen Presenting Cells

Despite the original idea that MAIT cells are antibacterial and not activated by viruses,^2^, ^3^ it is now clear that viruses can also activate MAIT cells by stimulating cytokine production through ligation of TLRs or other pattern recognition receptors.

Early studies did not find evidence of viral activation of MAIT cells.^2^, ^3^ Le Bourhis *et al*. assessed the ability of several viruses (encephalomyocarditis virus, Sendai virus, Newcastle disease virus, herpes simplex virus and parainfluenza 3 virus) to activate murine MAIT cells.^3^ While these viruses could activate bone marrow‐derived dendritic cells, they were unable to activate MAIT cells. Importantly, these experiments were performed with purified Vα19‐Vβ 6 transgenic T cells. MAIT cells from Vα19‐Vβ6 transgenic mice differ from human and nontransgenic mouse MAIT cells in that they are naïve and lack expression of the transcription factor PLZF.^39^, ^40^ PLZF is required for the expression IL‐12R and IL‐18R.^41^ Indeed, in PLZF^−/−^ mice, MAIT cells lack IL‐18R expression.^42^ Therefore, MAIT cells from the transgenic mice would not be expected to respond to virally induced cytokines due to the lack of PLZF expression and resultant lack of IL‐12R and IL‐18R.

Gold *et al*. found that human MAIT cell clones failed to respond to human monocyte‐derived dendritic cells infected with a replication‐deficient adenoviral vector or with vaccinia virus.^2^ Of note, 7/14 clones did not express CD161 and it was not determined in another two clones. CD161 expression is strongly associated with the level of IL‐18R expression.^16^ Therefore, IL‐18R expression may have been downregulated in the MAIT cell clones. In addition, many poxviruses, including vaccinia virus, encode functional IL‐18 binding proteins (IL‐18BP), which inhibit IL‐18 signalling.^43^, ^44^ Some replication‐deficient adenoviral vectors may also fail to elicit a strong innate response, including IL‐12 or interferon‐α production, from human monocyte‐derived dendritic cells.^45^ Therefore, the lack of MAIT cell activation in response to viral stimulation may represent a combination of reduced ability of the T cell clones to respond to IL‐12 and IL‐18, a lack of induction of cytokine production by the virus or active inhibition of cytokine signaling by the virus.

Van Wilgenburg *et al*. recently demonstrated that virus‐infected cells are able to activate MAIT cells.^25^ They found that dengue virus, influenza virus and hepatitis C virus were all able to activate MAIT cells *in vitro* in a cytokine‐dependent manner. Monocyte‐derived dendritic cells infected with dengue virus activated MAIT cells which produced interferon‐γ, small amounts of TNFα and upregulated expression of CD69 and granzyme B. Similarly, macrophages exposed to influenza virus or to hepatitis C virus were able to stimulate MAIT cells to produce interferon‐γ and upregulate granzyme B expression. MAIT cell activation by dengue virus was dependent upon IL‐12 and IL‐18, while activation by influenza virus and hepatitis C virus was dependent upon IL‐18; in the case of hepatitis C virus, there was also a contribution from IL‐15 to MAIT cell activation, but only in combination with IL‐18. Importantly, all viruses stimulated IL‐18 production *in vitro*. Type I interferons were also shown to contribute to MAIT cell activation by hepatitis C virus. Inhibiting interferons with B18R, an interferon‐α/β neutralizing protein, reduced MAIT cell production of interferon‐γ and upregulation of CD69 and granzyme B. None of the viruses were able to stimulate MAIT cells in the absence of myeloid cells; the presence of a myeloid cell was necessary for MAIT cell activation. Importantly, the supernatant from IL‐12‐ and IL‐18‐stimulated MAIT cells was able to inhibit hepatitis C virus replication and this depended upon interferon‐γ production. Therefore, release of cytokines and type I interferons by myeloid cells in response to viruses can activate MAIT cells, which in turn can potentially control viral replication.

Loh *et al*. found that influenza virus can activate MAIT cells via induction of cytokines.^46^ When PBMC were cocultured with influenza virus‐infected A549 cells, a lung epithelial cell line, MAIT cells produced interferon‐γ and upregulated CD69 and granzyme B expression. Consistent with the findings of van Wilgenburg *et al*., no activation was seen when sort‐purified MAIT cells were cultured with influenza virus‐infected A549 cells. A soluble mediator in the supernatant of PBMC cocultures was able to activate MAIT cells, and that MAIT cell activation could be partially blocked with an IL‐18 neutralizing antibody, but not with an IL‐12 neutralizing antibody. Depletion of monocytes from PBMC abrogated the response of MAIT cells to influenza virus‐infected A549 cells. Therefore, secretion of IL‐18 by monocytes is necessary for MAIT cell activation by influenza virus‐infected epithelial cells.

## Mait Cell Responses in Human Viral Infections

Changes in MAIT cell frequencies have been reported in a number of viral infections (Table 1). CD161^++^ CD8^+^ T cells were originally shown to be lost from the blood and the liver in chronic hepatitis C virus (HCV) infection.^11^ The number of MAIT cells in the liver, as defined by IL‐17‐producing CD8^+^ T cells, inversely correlated with the degree of liver fibrosis.^11^ Depletion of MAIT cells from the blood and liver in chronic HCV has subsequently been confirmed in other studies.^47^, ^48^, ^49^, ^50^, ^51^ One study found that the frequency of MAIT cells in the liver inversely correlated with the degree of inflammation and fibrosis, while another study found an inverse correlation with fibrosis in patients coinfected with HCV and HIV but not HCV monoinfection.^49^, ^51^ Increased expression of markers of activation and exhaustion has been reported on blood MAIT cells and markers of activation on liver‐derived MAIT cells.^47^, ^48^, ^50^, ^51^, ^52^ In chronically infected HCV patients, the response of both blood and liver MAIT cells to *E. coli* was impaired while the response to IL‐12 and IL‐18 or interferon‐α and IL‐18 was preserved^47^, ^49^, ^52^; in severe fibrosis, a reduction in interferon‐γ production by liver MAIT cells in response to IL‐12 and IL‐18 +/– *E. coli* was seen relative to mild fibrosis.^51^ While some reduction in activation marker expression was seen on blood MAIT cells post successful treatment of HCV with direct acting antiviral agents, their numbers and functional impairment to *E. coli* did not recover.^47^, ^49^, ^50^ Similarly, clearance of HCV reduced activation marker expression on liver MAIT cells but their response to *E. coli* remained functionally impaired; in contrast to blood, a significant increase in intrahepatic MAIT cell numbers was seen.^49^ In contrast, in patients treated with interferon, more blood MAIT cells expressed CD38 and produced less interferon‐γ in response to IL‐12 and IL‐18 at weeks 4 and 12 of treatment; CD38 expression returned to baseline by week 24 post completion of treatment, but the impaired response to IL‐12 and IL‐18 persisted.^52^ Similarly, in studies of patients treated with directly acting antiviral drugs with or without interferon, an impact of interferon‐α was seen in terms of MAIT cell activation over time *in vivo*.^25^

**Table 1:** Studies of human MAIT cells and viruses

Author/Year	Virus	Finding	Reference
Billerbeck/Kang *et al*. (2010)	HCV	CD161^++^/Tc17 cells reduced in blood and in liver with increasing fibrosis .	11
Cosgrove/Ussher *et al*. (2013)	HIV	MAIT cell depletion in the blood in early and chronic HIV, possibly through depletion rather than compartmentalization. No restoration with ART.	53
Leeansyah *et al*. (2013)	HIV	MAIT cell depletion in chronic HIV—remaining cells highly activated and dysfunctional.	54
Wong *et al*. (2013)	HIV	MAIT cell depletion in blood in HIV and HIV/TB infection. Lower frequencies associated with both acute and chronic HIV. ART did not restore numbers.	57
Greathead *et al*. (2014)	HIV	MAIT cell recovery in tissue on ART.	62
Fernandez *et al*. (2015)	HIV	MAIT cell depletion early in HIV but retain function.	56
Eberhard *et al*. (2014)	HIV	MAIT cell depletion in blood and lymph node in HIV, independent of disease progression ‐ possibly due to over stimulation by microbial products and cytokines	58
Saeidi *et al*. (2015)	HIV	MAIT cell depletion in HIV and HIV/TB regardless of ART. Associated with increased PD1 and decreased CCR6 expression.	61
Gaardbo *et al*. (2015)	HIV	MAIT cell depletion and lack of recovery with ART.	63
Ussher *et al*. (2015)	HIV	MAIT cell depletion from blood confirmed using molecular probe for TCR.	55
Leeansyah *et al*. (2015)	HIV	MAIT cells from HIV+ patients exhibited abnormal T‐bet and Eomes expression patterns that correlated with the deficiency in cytotoxic capacity and cytokine production. Patient's plasma IL‐7 levels correlated with frequency and functionality. *In vitro* stimulation of MAIT cells with IL‐7 restored effector functions, including cytotoxicity.	29
Vinton *et al*. (2016)	SIV	MAIT cell systemic depletion in SIV model.	64
Barathan *et al*. (2016)	HCV	MAIT cell frequencies decreased, activation (HLA‐DR, CD38) and markers of exhaustion (PD‐1, TIM‐3, CTLA‐4) and senescence (CD57) increased in chronic HCV.	48
Spaan *et al*. (2016)	HIV, HCV	MAIT cell depletion in both HIV and HCV. CD38 levels highest in coinfected patients with acute HCV. Interferon (IFN)‐α therapy in chronic HCV infections led to further decline in MAIT cell numbers. Low numbers persisted even after successful treatment (both IFN‐ and non‐IFN‐based treatments).	52
Eberhard *et al*. (2016)	HIV, HCV	MAIT cell depletion in blood and in liver.	50
Khaitan *et al*. (2016)	HIV	MAIT cell depletion in children and recovery with age/ART; early treatment associated with the best recovery.	59
Hengst *et al*. (2016)	HCV	MAIT cell depletion and dysfunction in HCV. Remaining peripheral MAIT cells showed an activated phenotype (granzyme B^+^, HLA‐DR^+^, PD‐1^+^ and CD69^+^). Dysfunction continued even after viral clearance.	47
van Wilgenburg/Scherwitzl *et al*. (2016)	HCV, dengue, Influenza	MAIT cell frequencies increased in acute dengue, but reduced in influenza and HCV infections. No recovery in HCV even after successful treatment. In all infectious settings, MAIT cells displayed markers of activation (CD38, HLA‐DR, granzyme B), which decreased on resolution of infection. During dengue virus infection, CD38 expression increased over the course of infection, peaking at the day of defervescence; CD38 expression was higher on patients with more severe disease. Role for IFN‐α and IL‐15 in activating MAIT cells.	25
Loh *et al*. (2016)	Influenza	Reduced MAIT cells frequencies in patients’ hospitalized with fatal H7N9 infection. Influenza‐exposed monocytes were able to induce granzyme B and IFN‐γ expression by MAIT cells, which was IL‐18 dependent.	46
Paquin‐Proulx *et al*. (2017)	HTLV‐1	MAIT cell depletion and functional impairment, but high expression of activation markers CD38 and HLA‐DR, in HTLV‐1 infection.	67
Beudeker *et al*. (2017)	HIV/HCV	MAIT cell depletion and dysfunction in relation to liver fibrosis.	51
Freeman *et al*. (2017)	HIV	MAIT cell depletion despite ART.	60
Boeijen *et al*. (2017)	HBV	MAIT cells not depleted from the periphery, but display activation markers that reduced with therapy.	65
Yong *et al*. (2017)	HBV	Decrease in polyfunctional MAIT cell frequencies, including IFN‐γ^+^ and IFN‐γ^+^ granzyme B^+^ cells, in HBV infection.	66
Bolte *et al*. (2017)	HCV	Intrahepatic MAIT cells frequencies were decreased during HCV infection, which was inversely correlated with liver inflammation. Intrahepatic MAIT cells during infection display an activated and cytotoxic phenotype. Treatment resulted in reduction in activation and increase frequency.	49

MAIT cells are also depleted from the blood in HIV infection.^53^, ^54^ Depletion of MAIT cells is seen in adults early in infection and persists in chronic infection, even in elite controllers^50^, ^53^, ^54^, ^55^, ^56^, ^57^, ^58^; currently, there is limited data on the fate of MAIT cells in acute infection and further data is required.^57^ It is not reversed with antiretroviral therapy.^53^, ^54^, ^57^, ^58^ MAIT cell depletion is also seen in children infected prenatally; in contrast to adults, the levels of MAIT cells gradually increase with antiretroviral therapy, with more recovery the younger antiretroviral therapy is initiated.^59^ In patients coinfected with HIV and HCV, those with severe fibrosis or cirrhosis have even lower levels of MAIT cells in blood.^51^ While it has been suggested that downregulation of CD161 may account for the apparent depletion of MAIT cells,^54^, ^60^ studies using the MR1 tetramer and molecular quantification of MAIT cells have confirmed MAIT cell depletion is involved.^55^, ^56^The remaining MAIT cells are activated and functionally impaired, with loss of cytolytic capacity and upregulation of markers of exhaustion^29^, ^54^, ^58^, ^61^, while decreased cytokine responses were seen when PBMC were treated with *E. coli*, MAIT cells, however, retained their ability to produce cytokines in response to ligand or PMA/ionomycin stimulation.^54^, ^56^ While some improvement in MAIT cell function is seen with antiretroviral therapy, cytolytic function only improves with IL‐7 treatment.^29^ The fate of MAIT cells in tissue is less clear. While MAIT cells are depleted from lymph nodes,^58^ there are conflicting reports on their fate in gastrointestinal mucosal tissues.^53^, ^54^, ^62^ MAIT cells do not accumulate in the liver in patients with HCV and HIV coinfection.^51^ Their loss from blood may reflect a combination of redistribution to gastrointestinal mucosal sites and activation‐induced cell death.^53^ Indeed, the frequency of MAIT cells in blood is inversely correlated with MAIT cell activation.^54^, ^58^ Increased tryptophan metabolism may also be associated with MAIT cell depletion in HIV.^63^

MAIT cells are also depleted in SIV‐infected rhesus macaques.^64^ Since this is an experimental model, it is possible to demonstrate that MAIT cells are depleted from peripheral blood, mesenteric lymph nodes and the lungs. Overall, in these experiments, there was evidence of activation and proliferation of remaining MAIT cells in SIV‐infected animals, suggesting increased turnover and loss of MAIT cells rather than tissue redistribution.^64^ Of note, this study was cross‐sectional; longitudinal studies in animal models of HIV infection are still required to define the fate of MAIT cells and the mechanism of their depletion.

MAIT cells have also been studied in chronic hepatitis B virus infection. In one study, the levels of MAIT cells in HBV were found to be no different in chronic infection compared to controls, although as in HCV and HIV the cells were more activated.^65^ Activation declined with treatment with entecavir and was lower in HBe antigen‐negative patients.^65^ Another study found a small reduction in circulating frequencies although a clear difference in granzyme B expression (as in HCV) and in overall functionality.^66^ There is quite some variability in the natural history of HBV and impact of therapy so likely further larger and longitudinal studies of different patient groups in different clinical settings will shed further light in this area.

MAIT cells are also depleted from the blood and show reduced functionality in patients infected with HTLV‐1.^67^ Increased expression of activation markers was seen, but the cells had reduced ability to produce interferon‐γ; similar to HIV infection, the frequency of MAIT cells in blood was inversely correlated with their expression of activation markers.^54^, ^58^, ^67^ In patients with HTLV‐I‐associated myelopathy/tropical spastic paraparesis, decreased expression of PLZF was noted. MAIT cell numbers were not associated with viral load and MAIT cells were not infected by HTLV‐1.^67^

In addition to blood‐borne and chronic infections, where there is now quite a large amount of observational data in cohorts, there is also recent data on acute infections, dengue and influenza virus. Two papers have studied patients infected with influenza (pandemic H1N1 and H7N9) and both showed an inverse correlation between MAIT frequencies and severity of disease.^25^, ^46^ One explanation for this may be a protective role for MAIT cells in this disease, although it may also be a consequence of infection—further *in vivo* studies and prospective studies are needed to define this further. In contrast, in dengue, where more severe disease is evident as dengue hemorrhagic fever, there was a temporal and quantitative association between activation of MAIT cells and onset of severe disease.^25^ Again, this activation may reflect the exaggerated pathology seen or potentially in this setting MAIT cells (along with other mediators) could be implicated in immune pathology. Resolution of MAIT cell activation (as defined by CD38 and granzyme B expression) was seen in the convalescent blood sample (collected at least 10 days after the onset of fever) from patients with dengue fever, although resolution was incomplete in the case of granzyme B. IL‐18 levels were also decreased in the convalescent sample, with a correlation between IL‐18 levels and IL‐18Ra expression on MAIT cells, and IL‐18Ra expression on MAIT cells and MAIT cell activation. There is no longitudinal data on the dynamic of MAIT cell activation in influenza virus infection.

Overall, there is an emerging body of work in clinical studies showing a depletion of MAIT cells to a greater or lesser extent in relation to acute and chronic infection and certainly examples to suggest this is not all due to redistribution to tissues. Similarly, under conditions of persistent activation, there are data emerging that the MAIT cell compartment is impacted with variable effects on function and phenotype measured. Underpinning this, there is a reasonable mechanistic model of how this may be occurring, with many gaps to fill in, but what is most missing is an understanding of the role of such TCR‐independent MAIT cell activation and/or depletion in clinical outcomes. Similarly, as the same pathways for activation can trigger other CD161^+^ populations, including subsets of γδ T cells, for example, the same issues also need addressing.^16^

## Consequences of Viral Activation of MAIT Cells

Virally triggered MAIT cells can express a number of functions which may be relevant to disease. The most obvious one, as indicated in the studies above, is secretion of interferon‐γ. This is an important antiviral cytokine and some viruses (such as HCV) respond very sensitively. Interferon‐γ expression can be triggered within a few hours of viral exposure *in vitro* and the majority of MAIT cells can be found to express this cytokine.^24^ Such early responsiveness of MAIT cells appears to be dependent on type I interferon (in combination as always), which may act on both the antigen presenting cells (APC) and the T cell. Virus/APC combinations which induce later T cell activation (after 12–24 h) appear to be more dependent on IL‐12/IL‐15/IL‐18 combinations. To what extent this is recapitulated *in vivo* is not yet known, but nevertheless, since most viruses will induce a strong type I interferon response, an early effect is predicted.

Other effector functions which can be measured include TNFα secretion, which can have antiviral functions such as inhibiting spread of HCV.^68^ MAIT cells also markedly upregulate expression of granzyme B upon activation, which can be used as an *in vivo* marker, but also potentially licenses the cell for cytotoxicity. Although MAIT cells express RORγt and can secrete IL‐17, this tends to be observed *ex vivo* only after very strong stimulation with PMA/ionomycin or after some culture. To what extent virally derived cytokine signaling could trigger IL‐17 is not known although potentially it could occur in appropriately “educated” MAIT cells where Type 17 responses had been effectively induced already. Tissue localization may be important in this “education” as MAIT cells in the female genital tract have recently been shown to be biased toward IL‐17 and IL‐22 production.^69^ There also remains an open question as to whether such responses can contribute to tissue inflammation and immunopathology as well as host defense. This issue was raised in studies of dengue, where the correlation between MAIT cell activation and more severe disease (dengue hemorrhagic fever) was observed.^25^ However, since correlation is not causation some care is needed here. HIV infection, for example, induces high levels of virus‐specific CD8^+^ T cell activation, which is much reduced in elite controllers, but there is also a great deal of evidence that CD8^+^ T cells can contribute to protection against progression.^70^ Thus, MAIT cell activation may be—like many effector responses—a two‐edged sword, with the overall outcome dependent on the dose and timing of infection, and the presence of other effector responses.

Beyond these functions, the impact of such triggering on MAIT cell proliferation and survival is also of interest. *In vitro* MAIT cells can respond to cytokine triggers through proliferation in the absence of a TCR signal.^30^ Thus, an important aim of experiments using animal models should be to test this *in vivo*. However, the most common finding in human studies is a depletion of MAIT cells in blood. There are data to suggest that MAIT cells are susceptible to activation‐induced cell death and this is readily observed following engagement of bacterially loaded APCs.^53^, ^71^ Data from HIV indicate that MAIT cells are depleted in blood and those that are found express markers of exhaustion and dysfunction.^29^, ^54^, ^58^, ^61^ Such markers may represent a pathway toward full deletion as seen in the LCMV model.^72^ To what extent such cells can recover is not known, although recent data from LCMV suggest this is relatively difficult due to epigenetic changes that occur through persistent stimulation.^73^

## Unanswered Questions

So far, it appears clear from studies of patients and from *in vitro* experiments that MAIT cells can sense virus infection through a cytokine‐driven network (Figure 2). However, many questions remain about the biological significance of these observations. First, overall, there is as yet no clear evidence that MAIT cell activation driven by virus infection is protective for the host or in fact deleterious. What is needed to develop this further are close studies of experimental models where the MAIT cell response can be manipulated. More data on mouse models should emerge which will address this important point. Since in the setting of bacterial infections (e.g. with *Francisella tularensis* 74) the effect is as part of a team rather than as a sole effector, this paradigm is likely to apply to MAIT cell responses to viruses. Clearly, there are other populations with overlapping cytokine‐driven profiles, including invariant NK T (iNKT) and NK cells, so defining a nonredundant role may not be simple. However, such data do exist for iNKT cells in response to influenza, indicating a route for MAIT cell studies to pursue.^75^ One very important difference here is in the dominance of the responses in humans *versus* mice—so the differences in frequency and in maturation status might require careful modeling or interpretation.

A second question mark hangs over the specificity of such responses. Since MAIT cells can be activated in the absence of any APC by cytokines alone, it is theoretically possible that they could be activated without any local focus as to the infected target. The *in vitro* data, however, do indicate that the process is very efficient in the presence of the target cell or APC (possibly a concentration effect) and importantly such cells can provide not only cytokine signals but also surface costimulatory signals which can direct activity. The same theoretical issues apply to other cytokine‐sensitive populations such NK cells. However, direct *in vivo* or *in vitro* evidence that MAIT cells activated in a TCR‐independent manner can kill virally infected cells would be of interest. Alternatively, since such innate stimuli can license MAIT cells for cytotoxicity,^30^ they may contribute to immunopathology by “off target” effects.

Third, and related to the last point, to what extent MAIT cell activation could lead to direct antiviral effects (as indicated in the HCV studies) and how much MAIT cells act indirectly as early orchestrators of adaptive and other innate responses should still be defined. Again, this would be best addressed in an animal model, assuming the MAIT cells can be used in a setting which represents human infection. This issue applies equally to bacterially driven responses as to virally driven ones.

Finally, a very common finding in MAIT cell studies in the last few years is the loss of MAIT cell frequencies in blood. Some of this may be due to tissue compartmentalization although this is not yet well defined and actual depletion of cells may well contribute. This has been particularly well reproduced in studies of HIV—and whether such modulation can impact on other host defense functions dependent on MAIT cells (e.g. protection against mycobacterial infection) is not yet known. The recovery of MAIT cell populations following acute viral infection or after therapy of chronic viral infection appears to be rather slow in adults, so to what extent the diversity in MAIT cell frequencies that is observed in humans is driven by viral encounters in the past or acquisition of persistent viruses such as herpesviruses, is not yet known.

Clearly at this point, there are more questions than answers, but since in humans MAIT cells can represent a substantial fraction of circulating and tissue T cells, their contribution to the immune response in viral infection—for better or for worse or simply as bystanders—does demand further investigation.

## Conflicts of Interest

The authors have no conflicts of interest.
